# Anterior and posterior left ventricular aneurysm repair concomitant with off pump coronary artery bypass grafting

**DOI:** 10.1186/1749-8090-8-S1-O210

**Published:** 2013-09-11

**Authors:** I Duvan, M Kurtoglu, S Ates, BE Onuk, P Sungur, IS Karacan

**Affiliations:** 1 Department of Cardiovascular Surgery, Güven Hospital, Ankara, Turkey

## Background

Necrosis after myocardial infarction may cause aneurysm in the left ventricle. Disorders of contractility in the left ventricle due to aneurysm can result with congestive heart failure, ventricular arrhythmia and thromboemboli. This is a retrospective analysis of our clinical experience in the restoration of left ventricular aneurysm via modified linear closure during off pump coronary artery bypass grafting (OPCABG) surgery.

## Methods

Ninety five patients who had post infarction, unruptured, true left ventricular aneurysm were operated between August 2006 and June 2013 during OPCABG. None of them had thrombus in their left ventricular cavity. Age of the patients were 63,14 ± 8,46 (38-82 year) meanly. Seventy two of them were male (75.8 %). Ejection fraction was 32,58% ± 6,98% preoperatively. Fifty three patients had totally occluded left anterior descending artery. Dyspnea (47.7%), angina (82.9%) and ventricular arrhythmia (11.3%) were the major symptoms. Six patients had posterior and 89 patients had anterior left ventricular aneurysm.

## Results

Number of coronary artery bypasses were 2,21±1,1. LIMA, RIMA, radial artery and saphenous vein grafts were used. Mean operation time was 127,92±32,52 minutes. Average time of intubation was 5,6±4,5 hours, intensive care unit stay was 26,1±15,1 hours, hospital stay was 5,3±2,2 days. Supraventricular arrhythmia was seen in 20 patients, cardioversion was performed in 5. Positive inotrope agents were required for 11, ICD implantation for 5, IABP for 3 and ECMO for 1 patient. Three patients (3.1 %) were dead in hospital.

## Conclusion

The technique of anterior and posterior aneurysm repair via modified linear closure concomitant with OPCABG can be performed with low operative morbidity and mortality rates.

